# Editorial: Computational and integrative approaches for developmental biology and molecular evolution

**DOI:** 10.3389/fgene.2023.1252328

**Published:** 2023-07-14

**Authors:** Bruno César Feltes, Rodrigo Ligabue-Braun, Márcio Dorn

**Affiliations:** ^1^ Department of Biophysics, Institute of Biosciences, Federal University of Rio Grande do Sul, Porto Alegre, Brazil; ^2^ Department of Pharmacosciences, Federal University of Health Sciences of Porto Alegre, Porto Alegre, Brazil; ^3^ Department of Theoretical Informatics, Institute of Informatics, Federal University of Rio Grande do Sul, Porto Alegre, Brazil; ^4^ Center of Biotechnology, Federal University of Rio Grande do Sul, Porto Alegre, Brazil; ^5^ National Institute of Science and Technology: Forensics, Porto Alegre, Brazil

**Keywords:** systems biology, bioinformatics, omics, machine learning, developmental biology, evolution

## 1 Introduction

Developmental Biology is a broad field where each type of biological data at the molecular, tissue, or phenotype level is pertinent to uncover an organism’s origins and developmental process. The final panorama is that each layer of information requires distinct mindsets for accurate data interpretation.

**FIGURE 1:**
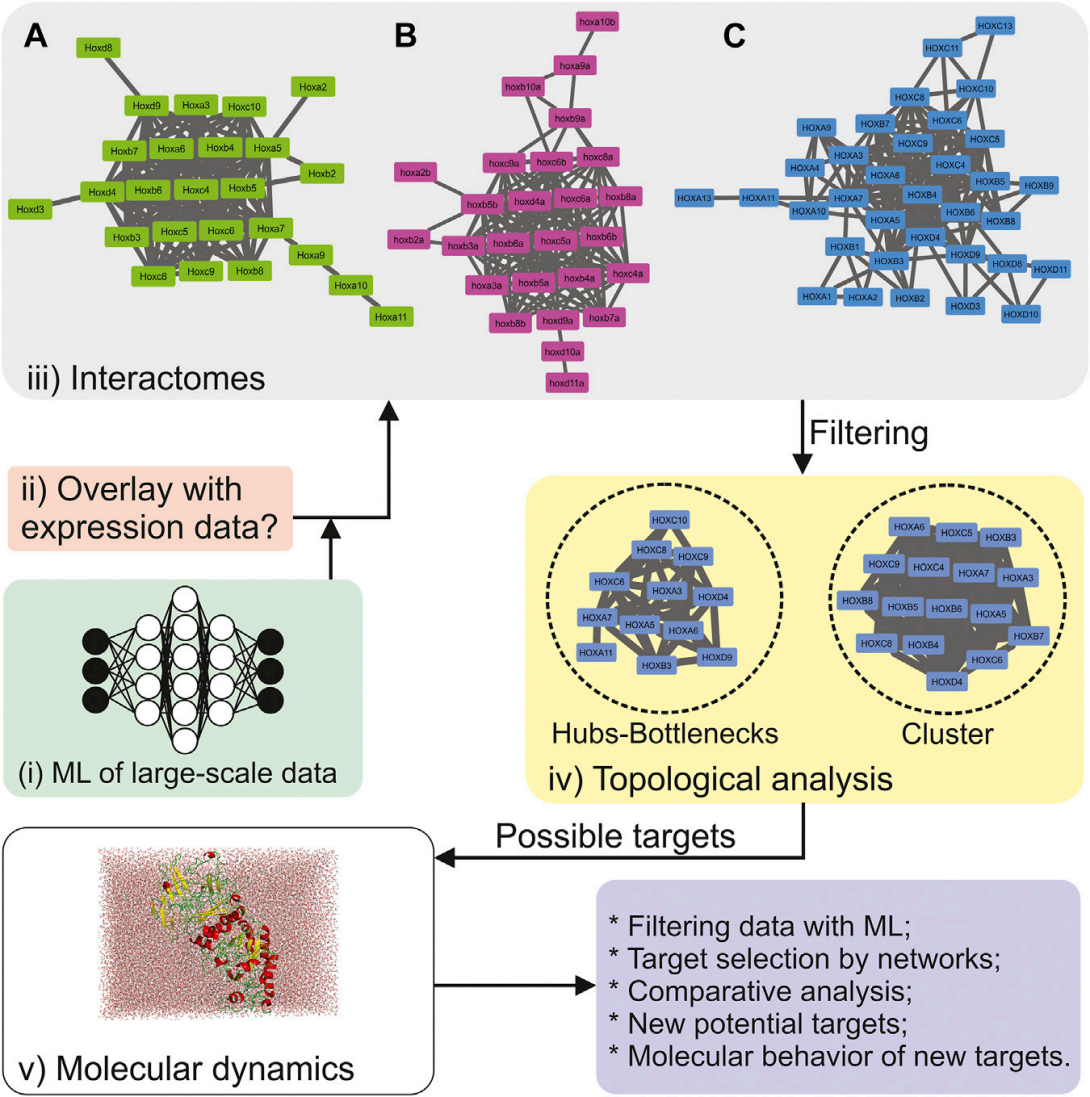
Example of an integrative computational approach to explore biological data. The concept is to integrate different computational approaches into one single pipeline. **(A)** Protein-Protein Network (PPN) for *Mus musculus*. From the 39 Hox proteins, Hoxa1, Hoxa4, Hoxa13, Hoxb9, Hoxb13, Hoxc11, Hoxc12, Hoxc13, Hoxd1, Hoxd10, Hoxd11, Hox12, and Hoxd13 were not connected to the network; **(B)** PPN for the 36 hox genes in *Danio rerio*. Hoxa1, hoxa11, hoxa13, hoxb1, hoxc10, hoxd3, hox12, and hoxd13 were not connected to the network, whereas hoxa9 and hoxc3 were not found by STRING; **(C)** PPN for *Homo sapiens*. From the 39 HOX proteins, HOXB13, HOXC12, HOXD1, HOXD12, and HOXD13 were not connected to the network. The primary networks were built in the STRING v.11 (Szklarczyk et al., 2019) meta-search engine, using the following parameters (10 Oct 2020): all search options enabled, except for *textmining* and gene-fusion, the confidence score of 0.4, and no more than 20 interactions in the 1st shell. The final networks were edited in Cytoscape 3.6.1. (Shannon, 2003). Clusters were obtained using MCODE (Bader and Hogue, 2003), and centralities were calculated by CentiScaPe 2.2 (Scardoni et al., 2009). Protein image was created using Pymol (DeLano, 2002), system was build using GROMACS 2018.1 (Abraham et al., 2015).

As Theodosius Dobzhansky expressed, “*Nothing in biology makes sense except in the light of evolution*,” (Dobzhansky, 1973) and Developmental Biology flawlessly reflects Dobzhansky’s statement. Because every piece of evolutionary characteristics are relevant to understanding an organism’s developmental process, it is common to use model organisms to compare data or look for indications to best invest time, resources, and efforts. When a molecular process is conserved in multiple organisms throughout evolution, the probability of being essential for organism survival increases. Therefore, multiple data layers are required to rigorously understand a developmental process. The final panorama is an escalation of data complexity. As complexity increases, the need to apply computational approaches grows proportionally. There are numerous ways to apply computational methods to Developmental Biology (Figure 1), each adapted to the question guiding the study and the type of data available. The same is true for diseases that take place during the developmental process.

For example, Network Systems Biology (NSB) is one of the most interdisciplinary subareas of Bioinformatics. NSB became an invaluable tool for understanding biological systems, for the formulation of original hypotheses, for comparison between different organisms, and for interpreting a massive amount of data (Vidal et al., 2011). This possibility arises because network topological proprieties and organization principles, such as clustering, percolation, and centralities, can be applied to numerous biological problems, regardless of the background study (Albert, 2005). However, despite this flexibility, how they can or should be utilized differs from data to data (e.g., gene expression, genomics, proteomics, epigenomics, etc.). Since the significant limitation of NSB is data availability, the continuous effort to create new databases, specially curated ones, directly impacts NSB studies’ success. New network topological parameters are also necessary for a more accurate selection of potential targets.

*In silico* structural analysis at the protein level is also a common integrative approach, combined with data from the wet lab or as a stand-alone analysis. Analyzing a single protein structure can be considered an entire universe of complexity because numerous investigations need to be applied to infer a molecular behavior or a possible structural feature. In this sense, there is a demand for more computational methods to analyze proteins essential to the developmental process. Due to the numerous limitations of experimental techniques at the wet lab, many proteins, such as HOX proteins, have no complete structural data. Therefore, the precision of protein structural modeling tools, especially using *ab initio* calculations, significantly increases the success of protein structure research (Delarue and Koehl, 2018).

Some widespread computational approaches, such as gene expression analyses and phylogenetic studies, are also essential in developmental biology research. Although the applicability of these studies is straightforward, they are not without challenges. Statistical analyzes are crucial in both cases, and the creation of new tools implementing robust statistical treatments for multi-level and meta-analyzes are decisive in generating accurate data. In particular, phylogenetic studies need new tools to deal with massive amounts of sequence.

Finally, machine learning (ML) approaches are gaining territory in almost all biological and Biomedical Sciences. Such tools’ vast applicability can be adapted to virtually all Big-Data problems. Either being applied to expression (Ang et al., 2016), genomic (Libbrecht and Noble, 2015), epigenetics (Holder et al., 2017) or biomedical image data (Kan, 2017), new ML algorithms and elegant application protocols are necessary for this day and age of science. In addition to developing new algorithms, the major challenge of this field is how they are tested. Input data is perhaps the major player in this equation since training the algorithm with up-to-date curated quality data would, undoubtedly, generate more accurate results than training them with older datasets (Feltes et al., 2019; Feltes et al., 2021).

This Research Topic presents some integrative approaches applied to developmental biology experiments from different backgrounds.

## 2 The research topic

Fagny et al. employed systems biology to integrate genomic, transcriptomic, and epigenomic data to elucidate the regulatory relationships between transcription factors, enhancers, and potential target genes in maze and identify regulatory key factors specific to leaves at the seedling stage and husks at flowering. By combining different omic data, they reconstructed tissue-specific-associated transcriptomic factors regulatory networks and uncovered genes crucial to tissue-specific differentiation.

Zhou et al. combined two RNA-seq datasets of human placentas from term and preterm birth to investigate co-altered circadian transcripts-associated long intergenic non-coding RNAs (lincRNAs). Using a diverse set of transcriptomic analyses, they uncovered nine core molecular clock genes deregulated by the decrease of five circadian lincRNAs in the placenta, affecting a myriad of crucial biological processes that could be linked to preterm birth.

Shang et al. analyzed cardiac development-associated transcriptomic data from three Single-Cell Tagged Reverse Transcription (STRT-Seq) datasets. Using a transcriptomic analysis workflow, they explored lineage-specific changes in mouse and humans regarding gene expression, subpopulation composition, and developmental features in cardiac tissues. They described an evolutionary conservation of cell populations and molecular profiles during heart development in both species.

Tang et al. reviewed experimental technologies, public data, and predictive models associated with synthetic lethal pairs. The knowledge of synthetic lethal pairs is deeply interconnected to developmental biology since it revolves around how the impairment of two genes can lead to cellular or organism death, which does not happen when one of these genes is still viable. The authors outlined important perspectives and critical discussions on the subject that can aid future research.

Finally, Wu et al. provided a comprehensive review of the mathematical model of local ancestry inference (LAI) applied to genomic data. In this sense, its application, historical aspects, different benchmarks, and the strengths and limitations of LAI are discussed and outlined.
